# Diagnoses and procedures of inpatients with female genital mutilation/cutting in Swiss University Hospitals: a cross-sectional study

**DOI:** 10.1186/s12978-022-01411-z

**Published:** 2022-05-02

**Authors:** Mathilde Horowicz, Sara Cottler-Casanova, Jasmine Abdulcadir

**Affiliations:** 1 Faculty of Medicine, University of Geneva, Geneva, Switzerland; 2 Division of Gynaecology, Department of the Woman, The Child and the Adolescent, Geneva University Hospitals, 30 Bld de la Cluse, 1211 Geneva, Switzerland; 3 Swiss Tropical and Public Health Institute, University of Basel, Basel, Switzerland

**Keywords:** Female genital mutilation, Female genital cutting, Female genital mutilation/cutting, International classification of diseases, ICD, Coding, Switzerland

## Abstract

**Background:**

Female genital mutilation/cutting (FGM/C) can result in short and long-term complications, which can impact physical, psychological and sexual health. Our objective was to obtain descriptive data about the most frequent health conditions and procedures associated with FGM/C in Swiss university hospitals inpatient women and girls with a condition/diagnosis of FGM/C. Our research focused on the gynaecology and obstetrics departments.

**Methods:**

We conducted an exploratory descriptive study to identify the health outcomes of women and girls with a coded FGM/C diagnose who had been admitted to Swiss university hospitals between 2016 and 2018. Four of the five Swiss university hospitals provided anonymized data on primary and secondary diagnoses coded with the International Classification of Diseases (ICD) and interventions coded in their medical files.

**Results:**

Between 2016 and 2018, 207 inpatients had a condition/diagnosis of FGM/C. The majority (96%) were admitted either to gynaecology or obstetrics divisions with few genito-urinary and psychosexual conditions coded.

**Conclusions:**

FGM/C coding capacities in Swiss university hospitals are low, and some complications of FGM/C are probably not diagnosed. Pregnancy and delivery represent key moments to identify and offer medical care to women and girls who live with FGM/C.

*Trial registration*: This cross-sectional study (protocol number 2018-01851) was conducted in 2019, and approved by the Swiss ethics committee.

## Plain English Summary

Female genital mutilation/cutting (FGM/C) can result in short and long-term complications, which can impact physical, psychological and sexual health. Our objective was to obtain descriptive data about the most frequent health conditions and procedures associated with FGM/C among inpatients with a condition/diagnosis of FGM/C in Swiss university hospitals. We asked the Swiss university hospitals anonymized data of women and girls with a coded FGM/C diagnose who had been admitted between 2016 and 2018. Four of the five Swiss university hospitals provided the primary and secondary diagnoses coded with the International Classification of Diseases (ICD) and the interventions coded in their medical files. Only 207 inpatients had a condition/diagnosis of FGM/C. The majority was admitted either to gynaecology or obstetrics divisions. Some complications of FGM/C are probably not diagnosed. Pregnancy and childbirth represent key moments to care for and counsel a population that might not consult or be identified otherwise.

## Introduction

Female Genital Mutilation/Cutting (FGM/C) comprises all procedures involving partial or total removal of the external female genitalia without medical indication [1]. The World Health Organization (WHO) defines four main types of FGM/C (Table 1) [2]. 200 million women and girls have undergone the practice in 31 countries according to nationally representative household surveys, without counting female migrants with FGM/C who live high-income countries [3, 4]. According to estimates, almost 600,000 individuals living in the European Union are believed to have been exposed to ritual genital cutting (2016) [5], and in Switzerland, approximately 21,706 women and girls are estimated to have been exposed to this practice (2018) [6]. These estimates were obtained by indirect measures: multiplying the number of female migrants from an FGM/C practicing country with the FGM/C prevalence rate from the same country. This method does not account for regional and ethnic variations of the practice within countries, and does not include corrections for any changes in attitudes towards FGM/C, which have been described among migrants [7–11], nor include other female genital modifications such as female genital cosmetic surgeries. The actual prevalence of FGM/C among communities of migrants remains unknown [12, 13]. Recent studies conducted in the United Kingdom (UK) showed significantly fewer cases of FGM/C than expected among minors according to prevalence estimates [14, 15]. Nevertheless, the total number of women and girls who have undergone FGM/C is expected to grow in high-income countries because of increasing migration from countries where FGM/C prevalence remains high [16]. Although several interventions effectively promote the abandonment of FGM/C, many countries are simultaneously facing population growth, with consequent increase in the absolute number of girls exposed to FGM/C [17].

**Table 1:** Classification of FGM/C types and subtypes according to WHO [2]

Type I	Partial or total removal of the clitoral glans (the external and visible part of the clitoris, which is a sensitive part of the female genitals, with the function of providing sexual pleasure to the woman), and/or the prepuce/clitoral hood (the fold of skin surrounding the clitoral glans)
Type Ia	Removal of the prepuce/clitoral hood only
Type Ib	Removal of the clitoral glans with the prepuce/clitoral hood
Type II	Partial or total removal of the clitoral glans and the labia minora, with or without removal of the labia majora
Type IIa	Removal of the labia minora only
Type IIb	Partial or total removal of the clitoris and the labia minora
Type IIc	Partial or total removal of the clitoris, the labia minora and the labia majora
Type III (Infibulation)	Narrowing of the vaginal opening with the creation of a covering seal. The seal is formed by cutting and repositioning the labia minora, or labia majora. The covering of the vaginal opening is done with or without removal of the clitoral prepuce/clitoral hood and glans
Type IIIa	Removal and apposition of the labia minora
Type IIIb	Removal and apposition of the labia majora
Type IV	All other harmful procedures to the female genitalia for non-medical purposes, for example, pricking, piercing, incising, scraping and cauterization

It has been widely studied that FGM/C, particularly type III, can result in short and long-term complications, which can impact physical, psychological and sexual health [1]. Systematic reviews and meta-analyses show that female individuals with FGM/C are at higher risk of dyspareunia, genito-urinary complications, prolonged labour, episiotomies, and birth complications [18–21]. Frequently cited as a limitation, the lack of high-quality studies makes it difficult to reach consensus surrounding the association between FGM/C and caesarean section, infertility and HIV [18–20]. Depending on the study design, some of the available data about FGM/C complications and their clinical management may be subject to self-report and recall bias [22]. Inappropriate health management due to the lacking training surrounding FGM/C may further bias the existing data. To our knowledge, no study has yet described FGM/C complications and associated procedures using hospital inpatient data coded with the International Classification of Diseases (ICD).

We sought to describe the most frequent health conditions and procedures associated with FGM/C in inpatient women and girls identified from ICD diagnoses of FGM/C from five Swiss university hospitals.

## Materials and methods

This cross-sectional study (protocol number 2018-01851) was conducted in 2019, and approved by the Swiss ethics committee. We invited all five Swiss university hospitals (Geneva, Lausanne, Bern, Basel and Zürich) to provide anonymized data for all inpatient adult women and girls (< 18 years) with a nationality from any of the 30 FGM/C practicing countries [3] in addition to all inpatients who had a coded condition/diagnosis of FGM/C between January 1, 2016 and December 31, 2018. We did not include inpatients from the Maldives, where FGM/C has been recently reported [23], because no nationally representative survey was available when the study began. Please note that we talk about a “condition/diagnosis” of FGM/C as the ICD contains specific codes for FGM/C, which are also used to justify reimbursement of healthcare provided in case of need by health insurances. We also use the term condition, to acknowledge the fact that not all women and girls with FGM/C are sick.

In Swiss university hospitals, healthcare professionals record the diagnosis responsible for the hospitalization (primary diagnosis); eventual complications that arise during the patient’s hospital stay, as well as any additional diseases treated (secondary diagnoses) in the patients’ electronic medical charts. Professional coders in Switzerland code this information with the German Modification of the tenth edition of the ICD (ICD-10-GM), and interventions are coded with the Swiss Classification of Surgical Interventions (CHOP) [24].

We received the requested data from four university hospitals: Geneva (HUG), Lausanne (CHUV), Bern (Inselspital), and Zürich (USZ). The university hospital of Basel (USB) did not participate due to logistical difficulties in data provision. All data were then merged in a single database using STATA version 15.

The data for all inpatient women and girls from the 30 targeted FGM/C countries and all primary and secondary diagnoses of FGM/C coded between January 1, 2016 and December 31, 2018 was anonymized. The university hospital of Bern did not provide data on the interventions performed. Lausanne and Zürich provided CHOP codes of the interventions performed, and Geneva provided the name of the CHOP interventions. We analyzed all diagnoses and interventions in patients’ records with a coded primary or secondary diagnosis of FGM/C. We provided descriptive statistics with mean, ± standard deviation, and median for continuous variables, numbers by categorical variables. We compared all diagnoses from our sample with the FGM/C ICD “tip-sheet” for FGM/C associated health conditions (full methods available in another manuscript) [25]. We focused our analysis on the gynaecology and obstetrics divisions, where most of the inpatients with an FGM/C code were admitted.

The Swiss Federal Office of Public Health, the Swiss Network against Female Circumcision, and Caritas Switzerland funded the study. They had no role in study design, data collection and analysis, decision to publish, or preparation of the manuscript.

## Results

In four of the five Swiss university hospitals, 207 inpatients received a primary (n = 22, 10.6%) or a secondary (n = 185) diagnosis of FGM/C during the study period (Table 2). Of these 207 women and girls, 199 (96%, 89.4%) were admitted either to gynaecology or obstetrics divisions. The remaining women and girls were admitted to other departments (surgery, internal medicine, emergency, and paediatrics).

**Table 2:** Description of inpatients with a FGM/C (n = 207) as primary or secondary diagnosis between 2016 and 2018 followed in one of four Swiss university hospitals (Geneva, Lausanne, Bern and Zürich)

Variables	2016 (n = 42)	2017 (n = 69)	2018 (n = 96)
Center, n (%)			
Geneva	20 (47.6)	24 (34.8)	67 (69.8)
Lausanne	13 (31.0)	10 (14.5)	19 (19.8)
Bern	3 (7.1)	23 (33.3)	6 (6.3)
Zürich	6 (14.3)	12 (17.4)	4 (4.2)
Country of origin, n (%)			
Benin	0 (0)	0 (0)	1 (1.0)
Burkina Faso	1 (2.4)	2 (2.9)	0 (0)
Cameroon	1 (2.4)	0 (0)	0 (0)
Côte d’Ivoire	1 (2.4)	1 (1.5)	1 (1.0)
Egypt	0 (0)	0 (0)	5 (5.2)
Eritrea	12 (28.6)	37 (53.6)	36 (37.5)
Ethiopia	2 (4.8)	3 (4.4)	2 (2.1)
Guinea	0 (0)	0 (0)	6 (6.2)
Guinea-Bissau	0 (0)	0 (0)	2 (2.1)
Mali	0 (0)	0 (0)	1 (1.0)
Mauritania	0 (0)	0 (0)	1 (1.0)
Nigeria	1 (2.4)	1 (1.5)	3 (3.1)
Senegal	0 (0)	0 (0)	3 (3.1)
Somalia	14 (33.3)	18 (26.1)	22 (22.9)
Sudan and South Sudan	1 (2.4)	1 (1.5)	3 (3.1)
Unknown or other	9 (21.4)	6 (8.7)	10 (10.4)
Service, n (%)			
Gynaecology	13 (31.0)	12 (17.4)	9 (9.4)
Gynaecology or obstetrics^a^	1 (2.4)	23 (33.3)	6 (6.3)
Obstetrics	23 (54.8)	33 (47.8)	79 (82.3)
Others	5 (11.9)	1 (1.5)	2 (2.1)
Mean age at first visit (± SD, median)	30.7 (± 12.0, 27)	27.7 (± 6.1, 27.4)	29.8 (± 6.7, 30)
FGM/C type, n (%)			
Type I	3 (7.1)	13 (18.8)	10 (10.4)
Type II	8 (19.1)	16 (23.2)	33 (34.4)
Type III	21 (50.0)	33 (47.8)	39 (40.6)
Type IV	0 (0)	1 (1.5)	2 (2.1)
Unspecified or other	10 (23.8)	6 (8.7)	12 (12.5)

^a^Data obtained from Bern did not specify whether patients were admitted in gynecology or obstetrics

The primary diagnoses of women with a secondary diagnosis of FGM/C (n = 185) spanned 11 chapters of the ICD-10 (Table 3). 156 inpatients had a primary diagnosis related to pregnancy and childbirth. The most frequent diagnoses were perineal laceration during delivery (n = 29, 18.6%), labour and delivery complicated by fetal heart rate anomaly (n = 16, 10.3%), prolonged second stage of labour (n = 13, 8.3%) and premature rupture of membranes (n = 13, 8.3%). Nine patients were admitted for some type of anaemia: anaemia complicating pregnancy, childbirth and the puerperium (n = 5), iron deficiency anaemia (n = 3), and post-haemorrhagic anaemia (n = 1). Primary diagnoses of genitourinary diseases included vulvar cysts (n = 4), and infectious diseases such as abscess of vulva (n = 2), chronic salpingitis and oophoritis (n = 1) and pyonephrosis (n = 1).

**Table 3:** Primary diagnoses of inpatients with a secondary diagnosis of FGM/C (n=185) presented by chapter of the ICD-10

Variables		N
ICD-10 chapter and codes	ICD-10 diagnoses	
Neoplasms		3
C77.4, C90.00	Malignant neoplasms	2
D25.9	Leiomyoma of uterus, unspecified	1
Diseases of the blood and blood-forming organs and certain disorders involving the immune mechanism		4
D50.8, D50.9	Iron deficiency anemias	3
D62	Acute posthemorrhagic anemia	1
Endocrine, nutritional and metabolic diseases		1
E55.9	Vitamin D deficiency, unspecified	1
Diseases of the circulatory system		1
I05.0	Rheumatic mitral stenosis	1
Diseases of the skin and subcutaneous tissue		1
L02.2	Cutaneous abscess, furuncle and carbuncle of trunk	1
Diseases of the genitourinary system		20
N13.1	Hydronephrosis with ureteral stricture, not elsewhere classified	1
N13.6	Pyonephrosis	1
N39.3	Stress incontinence	1
N70.1	Chronic salpingitis and oophoritis	1
N76.4	Abscess of vulva	2
N84.0	Polyp of corpus uteri	1
N90.7	Vulvar cyst	4
Pregnancy, childbirth and the puerperium		156
O00.1	Tubal pregnancy	1
O02.1	Missed abortion	1
O09.6	“Duration of pregnancy 37 to 41 completed weeks, 253 to 287 completed days”	4
O09.7	“Duration of pregnancy More than 41 completed weeks More than 287 completed days”	1
O12.1	Gestational proteinuria	1
O14.0, O14.1, O14.9	Pre-eclampsia	4
O24.0	Pre-existing type 1 diabetes mellitus, in pregnancy, childbirth and the puerperium	1
O24.4	Gestational diabetes mellitus	8
O30.0	Twin pregnancy	1
O32.1	Maternal care for breech presentation	1
O33.5	Maternal care for disproportion due to unusually large fetus	2
O34.2	Maternal care due to uterine scar from previous surgery	3
O34.30	Maternal care for cervical incompetence, unspecified trimester	1
O34.7	Maternal care for abnormality of vulva and perineum	1
O36.5	Maternal care for known or suspected poor fetal growth	4
O36.6	Maternal care for excessive fetal growth	2
O41.0	Oligohydramnios	2
O41.1	Infection of amniotic sac and membranes	1
O42.0, O42.11, O42.12	Premature rupture of membranes, onset of labor within 24 hours of rupture	13
O43.21	Placenta accreta	1
O44.11	Complete placenta previa with hemorrhage, first trimester	2
O48	Post-term pregnancy	4
O60.1	Preterm labor with preterm delivery	2
O61.0	Failed medical induction of labor	2
O62.8	Other abnormalities of forces of labor	2
O63.0	Prolonged first stage (of labor)	4
O63.1	Prolonged second stage (of labor)	13
O64.8	Obstructed labor due to other malposition and malpresentation	1
O65.4	Obstructed labor due to fetopelvic disproportion, unspecified	1
O66.2	Obstructed labor due to unusually large fetus	1
O66.5	Attempted application of vacuum extractor and forceps	1
O68.0	Labour and delivery complicated by fetal heart rate anomaly	16
O68.2	Labour and delivery complicated by fetal heart rate anomaly with meconium in amniotic fluid	3
O70.0	First degree perineal laceration during delivery	13
O70.1	Second degree perineal laceration during delivery	11
O70.2	Third degree perineal laceration during delivery	1
O70.3	Fourth degree perineal laceration during delivery	2
O70.9	Perineal laceration during delivery, unspecified	2
O71.1	Rupture of uterus during labour	1
O72.0, O72.1	Third-stage haemorrhage	3
O75.6	Delayed delivery after spontaneous or unspecified rupture of membranes	1
O75.7	Vaginal delivery following previous caesarean section	2
O80	Single spontaneous delivery	3
O98.8	Other maternal infectious and parasitic diseases complicating pregnancy, childbirth and the puerperium	1
O99.0	Anaemia complicating pregnancy, childbirth and the puerperium	5
O99.1	Other diseases of the blood and blood-forming organs and certain disorders involving the immune mechanism complicating pregnancy, childbirth and the puerperium	1
O99.2	Endocrine, nutritional and metabolic diseases complicating pregnancy, childbirth and the puerperium	1
O99.8	Other specified diseases and conditions complicating pregnancy, childbirth and the puerperium	4
Congenital malformations, deformations and chromosomal abnormalities		1
Q50.5	Embryonic cyst of broad ligament	1
Symptoms, signs and abnormal clinical and laboratory findings, not elsewhere classified		1
R74.0	Elevation of levels of transaminase and lactic acid dehydrogenase [LDH]	1
Injury, poisoning and certain other consequences of external causes		1
S72.3	Fracture of shaft of femur	1
Factors influencing health status and contact with health services		18
Z37.0	Single live birth	4
Z65	Problems related to other psychosocial circumstances	1
Z91.70	Personal history of female genital mutilation, type unspecified	1
Z91.71	Personal history of female genital mutilation, type 1	1
Z91.72	Personal history of female genital mutilation, type 2	2
Z91.73	Personal history of female genital mutilation, type 3	9

The mean number of secondary diagnoses coded among women with a primary or secondary diagnosis of FGM/C was 2.59 (median 2, range 0–15), spanning 16 chapters of the ICD-10 (Table 4). There were 281 secondary diagnoses related to pregnancy and childbirth, including 114 codes describing duration of pregnancy (O09.1-O09.7, O48). Other frequent codes were perineal laceration during delivery (n = 21), prolonged second stage of labour (n = 8), and anaemia complicating pregnancy, childbirth and the puerperium (n = 24).

**Table 4:** Secondary diagnoses of inpatients with a condition/diagnosis of FGM/C presented by chapter of the ICD-10

Variables		N
ICD-10 chapter and codes	ICD-10 diagnoses	
Certain infectious and parasitic diseases		27
A39.0	Meningococcal meningitis	2
A60.9	Anogenital herpesviral infection, unspecified	1
B18.1	Chronic viral hepatitis B without Delta virus	1
B65.0	Schistosomiasis due to Schistosoma haematobium [urinary schistosomiasis]	1
B68.1	Taenia saginata taeniasis	1
B95.1	Streptococcus, group B, as the cause of diseases classified elsewhere	17
B95.91	Other specified gram-positive anaerobic, non-spore forming pathogens causing diseases, classified elsewhere	1
B96.2	*Escherichia coli* [*E. coli* ] as the cause of diseases classified elsewhere	2
B98.0	*Helicobacter pylori* [*H. pylori*] as the cause of diseases classified in other chapters	1
Diseases of the blood and blood-forming organs and certain disorders involving the immune mechanism		33
D50.0	Iron deficiency anemia secondary to blood loss (chronic)	9
D50.8, D50.9	Iron deficiency anemias	10
D52.9	Folate deficiency anemia, unspecified	1
D57.1	Sickle-cell disease without crisis	1
D62	Acute posthemorrhagic anemia	3
D64.8, D64.9	Anemia, other or unspecified	6
D68.4, D68.9	Coagulation defect	2
D90	Immune compromise after radiation, chemotherapy and other immunosuppressive measures	1
Endocrine, nutritional and metabolic diseases		16
E03.8, E03.9	Hypothyroidism	6
E11.60	Type 2 diabetes mellitus with other specified complications	1
E44.0	Moderate protein-calorie malnutrition	1
E53.8	Deficiency of other specified B group vitamins	1
E55.9	Vitamin D deficiency, unspecified	2
E66.00, E66.91	Obesity	2
E83.38	Other disorders of phosphorus metabolism and phosphatase	1
E87.6	Hypokalemia	2
Mental, Behavioral and Neurodevelopmental disorders		3
F32.8	Other depressive episodes	1
F43.1	Post-traumatic stress disorder (PTSD)	1
F53.8	Other postpartum mental and behavioral disorders, not elsewhere classified	1
Diseases of the nervous system		3
G01	Meningitis in bacterial diseases classified elsewhere	2
G57.2	Lesion of femoral nerve	1
Diseases of the circulatory system		5
I05.0, I07.1	Heart valve diseases	2
I10.90	Essential hypertension, unspecified: No indication of a hypertensive crisis	1
I48.9	Unspecified atrial fibrillation and atrial flutter	1
I95.8	Other hypotension	1
Diseases of the respiratory system		1
J90	Pleural effusion, not elsewhere classified	1
Diseases of the digestive system		5
K21.9	Gastro-esophageal reflux disease without esophagitis	1
K59.0	Constipation	1
K64.3	Fourth degree hemorrhoids	1
K66.1	Hemoperitoneum	1
K66.8	Other specified disorders of peritoneum	1
Diseases of the skin and subcutaneous tissue		2
L20.8	Dermatitis	2
Diseases of the musculoskeletal system and connective tissue		1
M54.2	Cervicalgia	1
Diseases of the genitourinary system		49
N06.8	Isolated proteinuria with other morphologic lesion	1
N13.6	Pyonephrosis	1
N18.9	Chronic kidney disease, unspecified	1
N39.0	Urinary tract infection, site not specified	1
N73.6	Female pelvic peritoneal adhesions	1
N80.3	Endometriosis of pelvic peritoneum	1
N83.8	Other noninflammatory disorders of ovary, fallopian tube and broad ligament	1
N87.0	Mild cervical dysplasia	1
N90.7	Vulvar cyst	1
N90.8	Other specified noninflammatory disorders of vulva and perineum	1
N90.80	Female genital mutilation, type unspecified	3
N90.81	Female Genital Mutilation, Type 1	3
N90.82	Female Genital Mutilation, Type 2	5
N90.83	Female Genital Mutilation, Type 3	16
N90.88	Other specified non-inflammatory diseases of the vulva and perineum (FGM, Unspecified or other)	6
N92.0	Excessive and frequent menstruation with regular cycle	2
N94.1	Dyspareunia	2
N94.4	Primary dysmenorrhea	1
N97.1	Female infertility of tubal origin	1
Pregnancy, childbirth and the puerperium		281
O08.1	Delayed or excessive haemorrhage following abortion and ectopic and molar pregnancy	1
O09.1	“Duration of pregnancy 5 to 13 completed weeks, 35 to 91 completed days”	2
O09.2	“Duration of pregnancy 14 to 19 completed weeks, 92 to 133 completed days”	4
O09.3	“Duration of pregnancy 20 to 25 completed weeks, 134 to 175 completed days”	2
O09.4	“Duration of pregnancy 26 to 33 completed weeks, 176 to 231 completed days”	6
O09.5	“Duration of pregnancy 34 to 36 completed weeks, 232 to 252 completed days”	3
O09.6	“Duration of pregnancy 37 to 41 completed weeks, 253 to 287 completed days”	72
O09.7	“Duration of pregnancy more than 41 completed weeks, more than 287 completed days”	15
O13	Gestational [pregnancy-induced] hypertension without significant proteinuria	1
O14.9	Unspecified pre-eclampsia	1
O16	Unspecified maternal hypertension	2
O24.1, O24.3	Pre-existing diabetes mellitus, in pregnancy, childbirth and the puerperium	4
O24.4	Gestational diabetes mellitus	6
O32.2	Maternal care for transverse and oblique lie	2
O33.4	Maternal care for disproportion of mixed maternal and fetal origin	3
O34.2	Maternal care due to uterine scar from previous surgery	4
O34.6	Maternal care for abnormality of vagina	3
O34.7	Maternal care for abnormality of vulva and perineum	3
O36.0	Maternal care for rhesus isoimmunization	2
O36.5	Maternal care for known or suspected poor fetal growth	1
O36.6	Maternal care for excessive fetal growth	1
O41.0	Oligohydramnios	2
O42.0, O42.11	Premature rupture of membranes, onset of labor within 24 hours of rupture	3
O43.20	Placenta accreta	2
O44.11	Complete placenta previa with hemorrhage, first trimester	1
O45.9	Premature detachment of the placenta, unspecified	1
O48	Post-term pregnancy	10
O60.1	Preterm labor with preterm delivery	1
O60.3	Preterm delivery without spontaneous labour	5
O61.0	Failed medical induction of labor	2
O62.1	Secondary uterine inertia	3
O63.0	Prolonged first stage (of labor)	2
O63.1	Prolonged second stage (of labor)	8
O64.1	Obstructed labour due to breech presentation	1
O64.8	Obstructed labor due to other malposition and malpresentation	1
O66.8	Other specified obstructed labor	2
O68.0	Labour and delivery complicated by fetal heart rate anomaly	2
O69.8	Labour and delivery complicated by other cord complications	1
O70.0	First degree perineal laceration during delivery	14
O70.1	Second degree perineal laceration during delivery	7
O71.3	Obstetric laceration of cervix	1
O71.8	Other specified obstetric trauma	2
O72.0, O72.1	Third-stage haemorrhage	9
O72.3	Postpartum coagulation defects	1
O73.0	Retained placenta without haemorrhage	1
O73.1	Retained portions of placenta and membranes, without haemorrhage	1
O75.7	Vaginal delivery following previous caesarean section	3
O85	Puerperal sepsis	1
O86.2	Urinary tract infection following delivery	1
O87.2	Haemorrhoids in the puerperium	1
O90.2	Haematoma of obstetric wound	1
O98.3	Other infections with a predominantly sexual mode of transmission complicating pregnancy, childbirth and the puerperium	1
O98.4	Viral hepatitis complicating pregnancy, childbirth and the puerperium	1
O98.8, O98.9	Maternal infectious and parasitic diseases complicating pregnancy, childbirth and the puerperium	2
O99.0	Anaemia complicating pregnancy, childbirth and the puerperium	24
O99.2	Endocrine, nutritional and metabolic diseases complicating pregnancy, childbirth and the puerperium	7
O99.3	Mental disorders and diseases of the nervous system complicating pregnancy, childbirth and the puerperium	2
O99.6	Diseases of the digestive system complicating pregnancy, childbirth and the puerperium	2
O99.7	Diseases of the skin and subcutaneous tissue complicating pregnancy, childbirth and the puerperium	2
O99.8	Other specified diseases and conditions complicating pregnancy, childbirth and the puerperium	12
Symptoms, signs and abnormal clinical and laboratory findings, not elsewhere classified		3
R30.0	Dysuria	1
R74.8	Abnormal levels of other serum enzymes	1
R82.3	Abnormal findings on cytological and histological examination of urine	1
Definition of HIV infection stages		2
U60.9, U61.9	HIV classification	2
External causes of morbidity		2
Y57.9	Drug or medicament, unspecified	1
Y84.9	Medical procedure, unspecified	1
Factors influencing health status and contact with health services		290
Z21	Asymptomatic human immunodeficiency virus (HIV) infection status	1
Z22.3, Z22.8	Carrier of other infectious diseases	17
Z25.8, Z27.3, Z27.4	Need for immunization against specified viral diseases	8
Z30.4	Surveillance of contraceptive drugs	1
Z34	Supervision of normal pregnancy	3
Z35.2, Z35.4, Z35.8	Supervision of high-risk pregnancy	4
Z37.0	Single live birth	91
Z37.2	Twins, both liveborn	1
Z59	Problems related to housing and economic circumstances	1
Z64.8, Z65	Problems related to certain psychosocial circumstances	4
Z86.1	Personal history of infectious and parasitic diseases	1
Z86.7	Personal history of diseases of the circulatory system	2
Z87.8	Personal history of other specified conditions	1
Z91.70	Personal history of female genital mutilation, type unspecified	17
Z91.71	Personal history of female genital mutilation, type 1	22
Z91.72	Personal history of female genital mutilation, type 2	47
Z91.73	Personal history of female genital mutilation, type 3	63
Z91.74	Personal history of female genital mutilation, type 4	3
Z92.1	Personal history of long-term (current) use of anticoagulants	2
Z94.0	Kidney transplant status	1

Among diseases of the genitourinary system, coded diagnoses featured vulvar cyst (n = 1), urinary tract infection (n = 1) and mild cervical dysplasia (n = 1). Other secondary diagnoses related to infections were Streptococcus group B (n = 17), possibly describing a carrier-state in pregnant women, and carrier of other specified bacterial or infectious diseases (n = 17), and asymptomatic HIV status (n = 1). Eight women required immunization against viral diseases such as measles, diphtheria, and other viral diseases.

Mental disorders and sexual health conditions were rarely coded as either primary or secondary conditions. “Problems related to psychosocial and/or economic circumstances” appeared five times as secondary diagnosis, and once as a primary diagnosis for a minor inpatient that was admitted in paediatrics. Out of the other four minors with a code of FGM/C (n = 5), another was admitted in paediatrics to undergo surgery for mitral valve stenosis, and the remaining two were admitted in gynaecology for surgical treatment of a vulvar cyst. The only minor inpatient with a primary diagnosis of FGM/C underwent defibulation and had secondary codes related to pregnancy.

In total, there were 62 primary and secondary diagnoses of anaemia in 36 patients admitted in gynaecology or obstetrics. Among them, six had third-stage haemorrhage, six a first- or second-degree perineal tear, and nine underwent caesarean section 27 of 135 patients admitted in obstetrics (19%), had a primary or secondary diagnosis of anemia complicating pregnancy and childbirth.

Several coded diagnoses in our sample might be possible long-term complications of FGM/C found in the FGM/C “tip-sheet” [25] (Table 5). The most frequently coded diagnoses (primary and secondary combined) were: perineal laceration during delivery (n = 50, 37.5% of FGM/C type III), prolonged second stage of labour (n = 21, 28.6% of FGM/C type III), postpartum haemorrhage (n = 12, 41.7% of FGM/C type III), and vulvar cysts (n = 5, 80% of FGM/C type III).

**Table 5:** Specific codes for long-term complications to FGM/C when FGM/C was coded as primary or secondary diagnosis

Variables		Primary diagnosis of FGM/C (n = 22)	Secondary diagnosis of FGM/C (n = 185)	Percentage of FGM/C Type III
ICD-10 chapter and diagnoses	ICD-10 code			
Certain infectious and parasitic diseases				
Human immunodeficiency virus (HIV) disease	B20-24	0	0	
Mental, Behavioral and Neurodevelopmental disorders				
Recurrent depressive disorder	F32-33	0	1	50% (n = 1)
Generalized anxiety disorder	F41.1	0	0	
Post-traumatic stress disorder	F43.1	0	1	100% (= 1)
Sexual dysfunction, not due to an organic condition	F52	0	0	
Diseases of the genitourinary system				
Cystitis	N30	0	0	
Urinary tract infection, site not specified	N39.0	0	1	100% (n = 1)
Other inflammation of vagina and vulva	N76	0	0	
Dysplasia of cervix uteri	N87	0	1	0% (n = 0)
Other specified non-inflammatory disorders of vagina	N89.8	0	0	
Vulvar cyst	N90.7	4	1	80% (n = 4)
Non-inflammatory disorder of vulva and perineum, unspecified	N90.9	0	0	
Dyspareunia	N94.1	0	2	50% (n = 1)
Dysmenorrhea, unspecified	N94.6	0	0	
Other specified conditions associated with female genital organs and menstrual cycle	N94.8	0	0	
Pregnancy, childbirth and the puerperium				
Prolonged second stage of labour	O63.1	13	8	28.6% (n = 6)
First degree perineal laceration during delivery	O70.0	13	14	29.6% (n = 8)
Second degree perineal laceration during delivery	O70.1	11	7	44.4% (n = 8)
Third degree perineal laceration during delivery	O70.2	1	0	100% (n = 1)
Fourth degree perineal laceration during delivery	O70.3	2	0	50% (n = 1)
Perineal laceration during delivery, unspecified	O70.9	2	0	0% (n = 0)
Obstetric high vaginal laceration	O71.4	0	0	
Other specified obstetric trauma	O71.8	0	2	50% (n = 1)
Obstetric trauma, unspecified	O71.9	0	0	
Postpartum haemorrhage	O72.0, O72.1	3	9	41.7% (n = 5)
Low forceps delivery	O81.0	0	0	
Other and unspecified forceps delivery	O81.3	0	0	
Vacuum extractor delivery	O81.4	0	0	
Single delivery by caesarean section	O82	0	0	
Disruption of perineal obstetric wound	O90.1	0	0	
Certain conditions originating in the perinatal period				
Birth trauma	P10-15	0	0	

Medical or surgical interventions were carried out in 110 (56,5%) patients with FGM/C: 47 interventions in Geneva, 42 in Lausanne and 22 in Zürich (Table 6). The most frequent obstetrical intervention was caesarean section (n = 29, 48.3% of FGM/C type III). 14 patients had an episiotomy (35.7% of FGM/C type III) and 15 required unspecified manual assistance during delivery (20% of FGM/C type III). The most frequent intervention aimed at treating complications of FGM/C was surgery of the clitoris (n = 11, 36.4% of FGM/C type III). In Geneva, four inpatients underwent defibulation.

**Table 6:** Main intervention reported among patients with FGM/C according to hospital

Variables	Geneva (n = 111)	Lausanne (n = 42)	Zürich (n = 22)	Percentage of FGM/C Type III
Obstetrical interventions				
Cerclage of the cervix	1	0	0	100% (n = 1)
Pharmaceutical induction of labour	0	1	0	100% (n = 1)
Manual assistance during delivery:				
With episiotomy and instrumentation With episiotomy only Unspecified	0 0 0	5 1 13	2 6 2	42.9% (n = 3) 28.5% (n = 2) 20% (n = 3)
Caesarean section	20	8	1	48.3% (n = 14)
Perineal tear repair	2	6	0	37.5% (n = 3
Curettage for retained placenta	1	1	0	0% (n = 0)
Gynaecological interventions				
Ovarian cyst excision	1	0	0	100% (n = 1)
Myomectomy	1	0	0	100% (n = 1)
Salpingectomy	1	0	1	50% (n = 1)
Interventions related to FGM/C				
Clitoral surgery	8	3	0	36.4% (n = 4)
Vulvar cyst excision	2	0	0	100% (n = 2)
Vulvar abscess incision and drainage	1	0	0	0% (n = 0)
Defibulation	4	0	0	100% (n = 4)
Interventions possibly related to FGM/C				
Hymenectomy	0	0	1	100% (n = 1)
Repair of vulva and perineum	0	0	5	100% (n = 5)
Incision of vulva and perineum	0	0	4	75% (n = 3)
Other interventions				
Femoral fracture repair	0	1	0	
Hematopoietic stem cell transplant	0	1	0	
Lymph node biopsy	0	1	0	
Mitral valvuloplasty	1	0	0	
Retrograde ureteropyelography	1	0	0	
Ureteral pigtail placement	1	0	0	
Transvaginal suspension for urinary incontinence	0	1	0	
Trunk abscess incision and drainage	1	0	0	

## Discussion

### Main findings

In four Swiss university hospitals, 207 inpatients had a primary (n = 22, 10.6%) or secondary (n = 185, 89.4%) diagnosis of FGM/C coded at admission between 2016 and 2018 [26]. As discussed in our related paper on Swiss university hospitals’ capacities of coding FGM/C, this was much less than expected when compared with the number of inpatients who could have undergone FGM/C based on their nationality and indirect estimates (n = 4947) [26]. Either fewer women than expected have undergone FGM/C, or healthcare professionals did not identify and/or record it, or professional coders failed to code FGM/C, resulting in suboptimal coding. Nearly all patients with a coded condition/diagnosis of FGM/C were admitted to an obstetrics and/or gynaecology division, and most of their primary and secondary diagnoses were related to pregnancy and delivery.

### Limitations and strengths

Limitations included the absence of participation from Basel; of interventions’ data from Bern; the exclusion of outpatients, which would inform on the health conditions treated and interventions performed (e.g. defibulation) in ambulatory care; and of non-university hospitals, where most deliveries of women in the cantons of Bern and Zürich occur (Tables 7, 8) [27–34]. Future studies could assess the prevalence of FGM/C and associated health outcomes in all hospitals, and study regional variations, such as in areas near asylum centres. Application of our method is mostly limited by undercoding of FGM/C, which most likely results from insufficient training about FGM/C [26]. Besides gynaecology and obstetrics, health professionals working in paediatrics, travel medicine, infectious diseases, primary care, and migrant health programmes, could benefit from such training.

**Table 7:** Number of deliveries between 2016 and 2018 according to center [27−33]

	2016	2017	2018
Geneva (HUG)	4101	4182	4213
Vaud (CHUV)	3230	3227	3375
Bern (Inselspital)	1810	1827	2004
Zürich (USZ)	2960	2971	2969

**Table 8:** Living births according to canton and nationality category of the mother [34]

	2016			2017			2018		
	Total	Swiss^a^	Foreigners^b^	Total	Swiss	Foreigners	Total	Swiss	Foreigners
Geneva (Geneva)	5361	2253	3108	5441	2350	3091	5353	2331	3022
Vaud (Lausanne)	8730	4401	4329	8686	4350	4336	10,145	5879	4266
Bern (Bern)	10,113	7038	3075	10,141	7115	3026	10,145	7189	2956
Zürich (Zürich)	17,051	9602	7449	17,070	9490	7580	16,919	9402	7517

^a^Infants born to women with a Swiss nationality

^b^Infants born to women without a Swiss nationality

This study’s main strength was the use of ICD-10 codes to identify health complications of FGM/C, an affordable and objective method, easily reproducible over time, and at national and international level, with good comparability of data. Impact of training, specific care, as well as financial costs resulting from health complications of FGM/C might also be assessed using ICD codes. They could be used in both diaspora and FGM/C high prevalence countries, as an alternative to the FGM/C cost calculator developed by WHO only for high prevalence countries [35].

### Interpretation

Women with FGM/C might consult, be admitted or referred more frequently when pregnant, resulting in better FGM/C coding in obstetrics divisions. Furthermore, Swiss basic health insurance covers most pregnancy-related costs, facilitating access to healthcare [36]. Obstetricians and gynaecologists routinely perform genital examinations and are more likely trained to diagnose FGM/C [26]. FGM/C is also more likely to be recorded in obstetrics charts, because it can influence childbirth [1]. For instance, UK’s report on FGM/C prevalence in the National Health System (NHS) showed that 1630 women and girls had a consultation where FGM/C was recorded between October and December 2020, with 74.9% of attendances in midwifery or obstetrical units [37]. Antenatal consultations provide major opportunities to identify and care for individuals with FGM/C who might not seek or receive medical attention otherwise [1, 38].

Meta-analyses including studies from FGM/C practicing countries, and diaspora countries showed that FGM/C was significantly associated with prolonged labour, perineal tears, episiotomy, and non-significantly associated with caesarean section [19, 20]. Obstetric outcomes coded in our study were mainly prolonged second stage of labour (n = 21) and perineal lacerations (n = 50) especially of first- or second-degree (90%). 29 inpatients required a caesarean section, 14 episiotomy, and 15 assistance during delivery. We were not able to calculate the prevalence of complications from FGM/C for several reasons. Our data was fully anonymized, and thus some records could potentially be returning patients, so we cannot know the exact denominator of pregnant women in our sample. Second, the study was cross-sectional, and some pregnant women might have delivered after the end of the study, leaving their birth outcomes unknown.

Among 85,990 deliveries in 2017 in Swiss medical institutions, 54.7% of women had a perineal tear mainly of first- or second-degree (94.7%); 32.3% a caesarean section; 11.1% an assisted delivery, and 17% an episiotomy [39]. Considering that at least 135 women were pregnant (135 inpatients admitted in obstetrics, and 30 in gynaecology and/or obstetrics), and subject to the limitations stated above, our data do not suggest high rates of obstetric complications.

Studies about obstetric complications of FGM/C sometimes show diverging results. A prospective study conducted in six African countries found a significant association between obstetric complications and FGM/C, especially type III [40], whereas retrospective studies from high-income countries such as Sweden, the UK, and Switzerland showed similar obstetric outcomes among women with and without FGM/C [41–43]. FGM/C has been significantly associated with higher rates of caesarean sections in studies conducted in both practicing and diaspora countries [40, 44, 45], and meta-analyses show a non-significant trend towards higher rates [19, 20]. Future studies could assess if training of health professionals and access to interpreters could improve obstetric outcomes of individuals with FGM/C. Indeed, health professionals unfamiliar with FGM/C might perform caesarean sections for inappropriate reasons, especially in cases of infibulation [46]. Moreover, migrant women in high-income countries often have higher rates of caesarean sections than non-migrants [47]. Communication barriers, economic difficulties, and exposure to violence can result in poor maternal health and/or care quality for some migrants regardless of FGM/C [48–52].

Only five minor inpatients had an FGM/C code. Outpatient clinics may attend more children with FGM/C than hospitals, but paediatricians may also not know when and how to discuss FGM/C with parents and their children, not recognize it if they perform a genital examination, or simply not record it [53–55]. Alternatively, they could be second-generation migrants and beyond, and therefore less exposed to the practice. A UK study showed that among 55 children with FGM/C referred to specialized clinics, 21% suffered from mental health symptoms such as anxiety, sleep and behaviour disorders, and 13% from physical symptoms such as problems with micturition, menstruation and genital pain [14]. Except one post-traumatic stress disorder, psychological symptoms were not coded in our minor population, and rarely among adults. Swiss university hospitals’ health professionals may lack time or training on how to detect and treat such symptoms and other FGM/C complications. Or, they may identify and manage psychological complications, without however identifying or documenting the FGM/C as an associated condition [54–60].

Coding of surgical interventions was incomplete. Perineal tears were more coded (n = 50) than perineal tears repairs (n = 8). Other repairs were either not coded, or coded as secondary interventions, which were not provided. Because no CHOP codes exist for defibulation and clitoral reconstruction, we had to hypothesize that codes such as repair (n = 5), or incision (n = 4) of vulva and perineum had been used to indicate these surgeries. Geneva provided the interventions’ names instead of codes, and reported 8 clitoral surgeries and 4 defibulations among inpatients, and additionally reported 12 clitoral surgeries, 25 defibulations and 8 other surgeries for scar complications of FGM/C in outpatient care. Some Swiss insurance companies have tried to refuse to reimburse these surgeries. Specific CHOP codes would facilitate medical coding and reimbursement.

Sensitisation and training of healthcare professionals and professional coders on FGM/C could improve identification, documentation and coding of FGM/C and its complications in Swiss university hospitals; inform and improve the quality of future policies, services and interventions. Future prospective and case–control studies could assess coding of FGM/C and associated health outcomes according to training and specialised care resources.

## Conclusion

Most of the 207 women and girls admitted to Swiss university hospitals between 2016 and 2018 with a primary or secondary diagnosis of FGM/C were admitted to obstetrics divisions. Pregnancy and delivery seem to be key moments to care for and counsel a population that might not consult or be identified otherwise. FGM/C coding capacities in Swiss university hospitals are low, and some complications of FGM/C are probably not diagnosed, or diagnosed alone, without FGM/C.

## Data Availability

The datasets used and/or analysed during the current study available from the corresponding author on reasonable request.

## Abbreviations

CHOP: Swiss Classification of Surgical Interventions
CHUV: University Hospital of Lausanne
FGM/C: Female genital mutilation/cutting
HUG: Geneva University Hospitals
ICD: International Classification of Diseases
NHS: National Health System
USB: University Hospital of Basel
USZ: University Hospital of Zürich
UK: United Kingdom
WHO: World Health Organization
